# Ion Pathways in Biomineralization: Perspectives on Uptake, Transport, and Deposition of Calcium, Carbonate, and Phosphate

**DOI:** 10.1021/jacs.1c09174

**Published:** 2021-12-09

**Authors:** Keren Kahil, Steve Weiner, Lia Addadi, Assaf Gal

**Affiliations:** ^†^Department of Chemical and Structural Biology and ^‡^Department of Plant and Environmental Sciences, Weizmann Institute of Science, 7610001 Rehovot, Israel

## Abstract

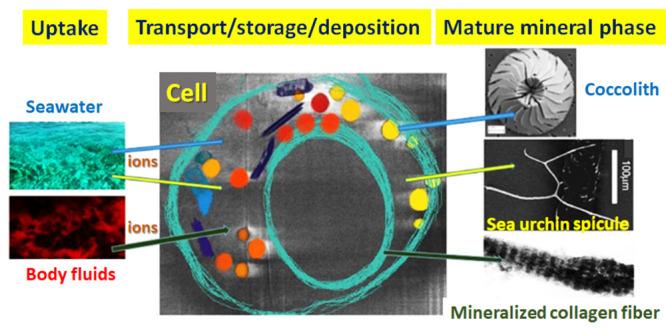

Minerals are formed by organisms in all of the kingdoms of life. Mineral formation pathways all involve uptake of ions from the environment, transport of ions by cells, sometimes temporary storage, and ultimately deposition in or outside of the cells. Even though the details of how all this is achieved vary enormously, all pathways need to respect both the chemical limitations of ion manipulation, as well as the many “housekeeping” roles of ions in cell functioning. Here we provide a chemical perspective on the biological pathways of biomineralization. Our approach is to compare and contrast the ion pathways involving calcium, phosphate, and carbonate in three very different organisms: the enormously abundant unicellular marine coccolithophores, the well investigated sea urchin larval model for single crystal formation, and the complex pathways used by vertebrates to form their bones. The comparison highlights both common and unique processes. Significantly, phosphate is involved in regulating calcium carbonate deposition and carbonate is involved in regulating calcium phosphate deposition. One often overlooked commonality is that, from uptake to deposition, the solutions involved are usually supersaturated. This therefore requires not only avoiding mineral deposition where it is not needed but also exploiting this saturated state to produce unstable mineral precursors that can be conveniently stored, redissolved, and manipulated into diverse shapes and upon deposition transformed into more ordered and hence often functional final deposits.

## Introduction

Many organisms form minerals for a wide variety of functions. This process of biomineralization involves many different minerals. About half of these minerals contain calcium, including the abundantly formed calcium carbonates (mainly calcite and aragonite, but also the less abundant vaterite and hydrated species). The predominant mineral of the vertebrate skeleton is the calcium phosphate mineral called carbonate hydroxyapatite. Nevertheless, all vertebrates also form calcium carbonate as part of their gravity and sound reception detectors.^1^ Calcium, carbonate, and phosphate are also integral components of the metabolism of every cell, serving fundamental roles in signal transduction and protein activity.^2^ Here, we provide some aspects of the uptake, transport, and deposition of these ions in biology from a chemical perspective. We do bear in mind the insightful adage that “biology is chemistry with a history”.^3^ This implies that we should be well aware that we cannot solve biological mysteries without considering chemistry. On the other hand, biology developed abilities during evolution to manipulate chemistry seemingly almost at will, in order to form minerals. These minerals are formed at the appropriate locations, at the correct time and rate, and with appropriate atomic order.

The ultimate source of ions is from the environment in which the organism lives. The process of biological mineral deposition can be divided into stages: ions must reach the tissue, combine at some stage with their counterions, achieve supersaturation of the salt solute, and finally precipitate as a solid phase. Whichever way the ions reach the mineral deposition site, be it from blood, seawater, or other media, these ions are concentrated by orders of magnitude when they are transformed from their dissolved state to the solid mineral. The calcium concentration is 10 mM in seawater, 1 mM in freshwater, and 27 M in calcite. The calcium concentration in vertebrate blood is 1–2 mM^4^ and in carbonate hydroxyapatite is 31 M. Therefore, all the calcium contained in 2.7 × 10^3^ mL of seawater is needed to deposit 1 cm^3^ of calcite and all the calcium contained in 3 × 10^4^ mL of blood is needed to deposit 1 cm^3^ of carbonate hydroxyapatite. At the same time, individual cells need to maintain their calcium signaling capability by reducing the calcium concentration in their cytosol to 100–200 nM. A major question therefore is: how do mineralizing organisms balance all these different requirements?

The calcite, aragonite, and carbonate hydroxyapatite that many organisms use for forming their mineralized tissues have very low solubilities. Seawater is supersaturated with respect to calcite and aragonite,^5^ and blood is supersaturated with respect to carbonate hydroxyapatite.^6^ There is thus a need to not only induce crystallization at the site of mineralization but also prevent precipitation where and when it is not desired. Moreover, ions are often temporarily stored.^1^ These ions should be stored in a state that enables them to be easily mobilized, namely, more soluble. In contrast, ions that are deposited in the final mineralized tissue product need to be in a solid state that produces a functional material. This is usually in the more dense crystalline state, but in some cases the mature mineral is in a disordered state. How is all this achieved?

A simple assumption is that the calcium and carbonate pathways are tailored to the needs of calcium carbonate depositors and the calcium and phosphate pathways to the needs of calcium phosphate depositors. The more we learn, however, the more we realize that this simple assumption is often violated. Many calcium carbonate depositors are manipulating aspects of their pathways with phosphate, and many calcium phosphate depositors are manipulating aspects of their system with carbonate.^7^ Yet another complication is that there may well be several complementary and/or redundant ion uptake, transport, storage and deposition pathways. Redundancy is a common phenomenon in biology. Despite all this complexity, the biological processes involved in manipulating calcium, carbonate, and phosphate and their corresponding biogenic minerals must obey the rules of thermodynamics (although often heavily influenced by kinetics) that determine the properties of these ions in solution and in a solid state.

The strategy we use in this Perspective is to first compare and contrast the ion pathways in three test cases. We chose calcite deposition in coccolithophores since these single celled organisms produce approximately half of the calcium carbonate deposits in the oceans.^8^ Coccolithophores are thus very efficient and very successful calcifiers. We chose spicule deposition in sea urchin larvae, because sea urchins are easily amenable to fertilization in the laboratory and therefore the process of mineral formation has been studied in detail.^9^ Finally, we chose to examine the pathways leading to carbonate hydroxyapatite deposition by vertebrates to form their bones. This pathway is probably the most widely investigated but also possibly the most complicated.

In the discussion, we try to identify possible common underlying processes used in all three systems, in the hope that at least some may also apply to other biomineralizing systems. In order to facilitate comparisons of such different systems, we divide the pathways into three stages: uptake and transport to the site of mineral formation, roles of the cells responsible for mineral formation, and processes involved in mineral deposition and maturation.

## Test Cases

### Coccoliths

**Figure 1:**
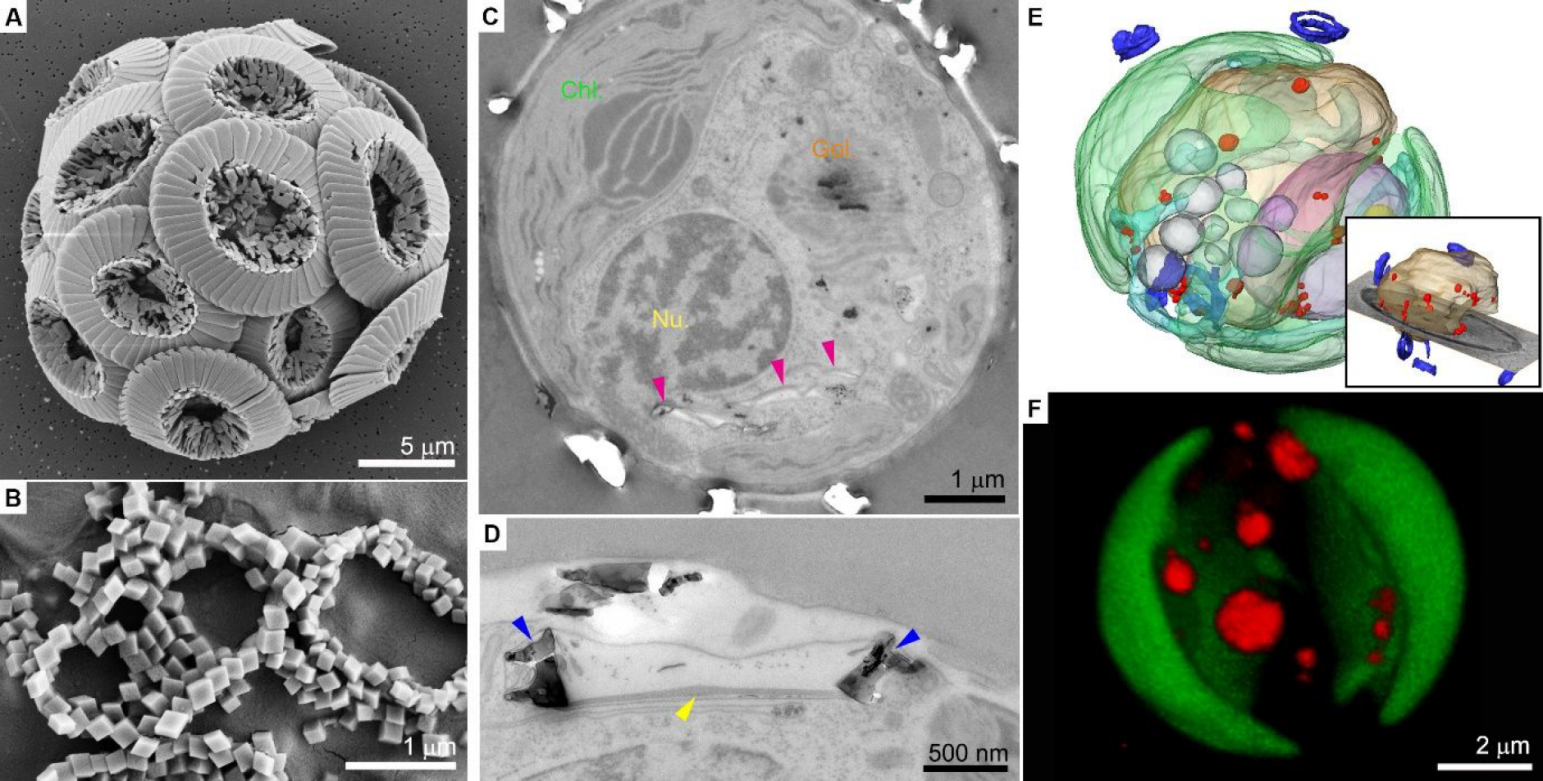
Formation of coccoliths. (A,B) SEM images of *Coccolithus braarudii* coccoliths. The diploid life stage forms intricate crystal morphologies (A), while the haploid stage forms simple rhombohedral crystals (B). (C–F) Cellular anatomy of *Pleurochrysis carterae*. (C, D) Sections in fixed cells show in (C) the various cell organelles (Chl., chloroplast; Nu., nucleus; magenta arrowheads indicate coccolith vesicles), and a high magnification image shows in (D) a coccolith vesicle (blue arrowheads indicate the crystals, yellow arrowhead indicates the organic base plate). Reprinted with permission from ref (22). Copyright 2020 Elsevier. (E) 3D rendering of a cryo-fixed cell. The inset shows the vacuole (light brown) filled with Ca–P-rich bodies (red) and coccoliths (blue). (F) 3D rendering of a live cell using confocal microscopy, showing several dense intracellular pools stained with DAPI (red); chloroplasts are in green. Reprinted with permission from ref (18). Copyright 2021 Wiley-VCH GmbH.

Coccoliths are mineralized scales that are produced intracellularly by coccolithophores, a group of marine unicellular algae.^10^ Each coccolith is an ordered array of calcite crystals and contains organic macromolecules (Figure 1A, B). The formation of the coccolith occurs inside a specialized organelle (the coccolith vesicle) and starts with the assembly of an organic base plate.^11^ Calcite crystals start to grow on this substrate, and when the coccolith is mature, it is exocytosed to the cell surface (Figure 1C, D).^12^ The crystals adopt a wide range of morphologies and architectures that are species-specific and their formation is under the strict control of the cell.^10,13^

The calcite crystals of coccoliths are very small, less than a micrometer in their thinnest dimension. Despite their small size, these calcite crystals show a high degree of crystallinity and contain very small concentrations of impurities and chemical substitutions.^14^ Their chemical purity, and specifically the very low levels of Mg substitution in the calcite lattice,^15^ demonstrate that many of the ions in seawater are excluded during the deposition process.

#### Uptake and Transport

The building blocks for coccolith crystallization are ions in solution that the cell extracts from the surrounding seawater. The chemical composition of modern seawater is dominated by Na^+^, Cl^–^, Mg^2+^, and SO_4_^2–^, whereas Ca^2+^ and bicarbonate (HCO_3_^–^) are less abundant with concentrations of ∼10 and ∼2 mM, respectively. Bicarbonate ions are the main carbonate species that are taken into the cell.^16^ Inside the cell, carbon utilization is divided between the need of CO_2_ for photosynthesis and the need of carbonate ions (CO_3_^2–^) for calcification.^16^ Even though these two processes are not functionally coupled, they represent two important requirements for carbon that need to be regulated by the cell in order to maintain homeostasis.

Ion transport from seawater to the coccolith vesicle is a highly selective process. Ion selectivity is mediated by various proteins that function as ion channels and pumps in the cell membrane. These complexes allow the passage of ionic species according to their molecular affinity to the transport machinery.^17^ At this initial stage, the cell discriminates against the prevailing Mg^2+^ ions in seawater in favor of calcium and creates a chemical composition inside the cell that is very different from seawater. Another important ion transport process is the expulsion of excess protons, formed during calcite precipitation, back to the environment.^8^ Calcium, bicarbonate, and proton transport pathways are part of the metabolism and signal transduction of every living cell, and the cellular machineries that evolved for homeostatic transport pathways may or may not be the same pathways used for mineralization. Importantly, direct uptake of seawater by endocytosis is not involved in coccolith formation,^18^ even though it is common in other biomineralization processes.^19^ This is demonstrated by the fact that small molecules with an affinity for calcium such as calcein, which cannot pass through membranes due to their charge, are not incorporated into coccolith calcite.^18^

#### Intracellular Ion Trafficking

The trafficking of ions for mineral formation inside this single celled organism is much more complex than previously assumed. The simplest scenario, often taken as a default in the absence of alternatives, is to think of the cell interior as an intermediate ion pool that transports ions to the terminal ion pool, the vesicle in which the coccolith is formed. In several studies, this assumption was extended to include additional ion pools within the cell that participate in the transport systems.^8^ These were usually carbon pools, such as the chloroplast, that affect carbonate speciation within the cell.

The trafficking of calcium is challenging for any eukaryotic cell, because of the need to maintain the concentration of Ca^2+^ in the cytoplasm at the sub-micromolar scale in order to avoid cytotoxic phenomena. Coccolithophores may circumvent this limitation by maintaining various intracellular compartments that store dense phases with high concentrations of calcium. One such compartment is a vacuole-like organelle that contains spherical condensates unexpectedly comprising primarily calcium and phosphorus and, not as would be expected, calcium and carbonate (Figure 1E,F). The concentration of calcium in these dense phases can reach ∼10 M. The exact chemical form of the phosphorus is not clear, but some indirect indications suggest that these are mainly polyphosphates.^7b,18^ These anatomical and chemical characteristics are reminiscent of acidocalcisomes, ion-rich organelles present in various organisms, most of which do not form any minerals.^21^ A second class of calcium-rich organelles in coccolithophores are compartments that contain various membranes, vesicles, and other cellular content.^22^ These ion-rich compartments have distinct anatomical properties and are characterized by a diverse chemical composition that can contain multiple elements such as Na, Mg, P, S, and Ca.^22^

Currently, we do not know the physiological roles of either of these ion-containing compartments. It was shown that under some conditions calcium can travel from these intracellular stores to the calcite crystals of the coccoliths,^23^ but other evidence shows that these compartments do not change in number, size, or abundance as a function of calcification activity.^18^ Therefore, the most accurate way to describe the current state of knowledge is that the coccolithophore cell contains several dense mineral phases (the coccolith vesicle, Ca–P-rich bodies, ion-rich compartments), which presumably maintain a complex net of interactions and may have diverse sets of functions. The compartmentalization into distinct pools within the cell allows flexibility in the use of calcium and carbonate extracted from seawater. Some of these pools may act as storage for calcium and phosphate, while others may transfer material for the formation of coccoliths.

#### Coccolith Formation

The calcite crystals of the coccolith nucleate and grow inside the confined environment of the coccolith vesicle (Figure 1D). The morphologies of the crystals are very diverse between species and between life stages. The morphologies range from the thermodynamically preferred rhombohedral habit of calcite to highly irregular and anisotropic structures (Figure 1A, B). Even in the more complicated shapes, it is usually possible to recognize some crystallographic facets.^14^ Importantly, the morphology at the nanometer scale of the crystal surfaces is smooth, lacking the granular texture that characterizes crystals formed via an intermediate amorphous phase.^24^ Recent native-state imaging of coccolith formation showed that precursor phases dissolve inside the coccolith vesicle, giving way to a process that is predominantly ion-by-ion growth and does not involve an amorphous calcium carbonate precursor phase.^25^

Nevertheless, the crystallization process involves conditions that are different from crystal nucleation and growth in bulk solution in vitro. Macromolecules play important roles in the confined environment of the coccolith vesicle. Charged polysaccharides complex calcium ions and transport them to the site of crystallization.^26^ Interactions between these polysaccharides and the organic base plate direct crystal nucleation to specific sites where dense polymer-calcium phases are formed.^26,27^ These chemical interactions between inorganic ions, soluble macromolecules, and insoluble organic scaffolds can direct the chemistry of this system to favor nucleation and growth of the calcite crystals under controlled conditions.

### Sea Urchin Larval Spicules

**Figure 2:**
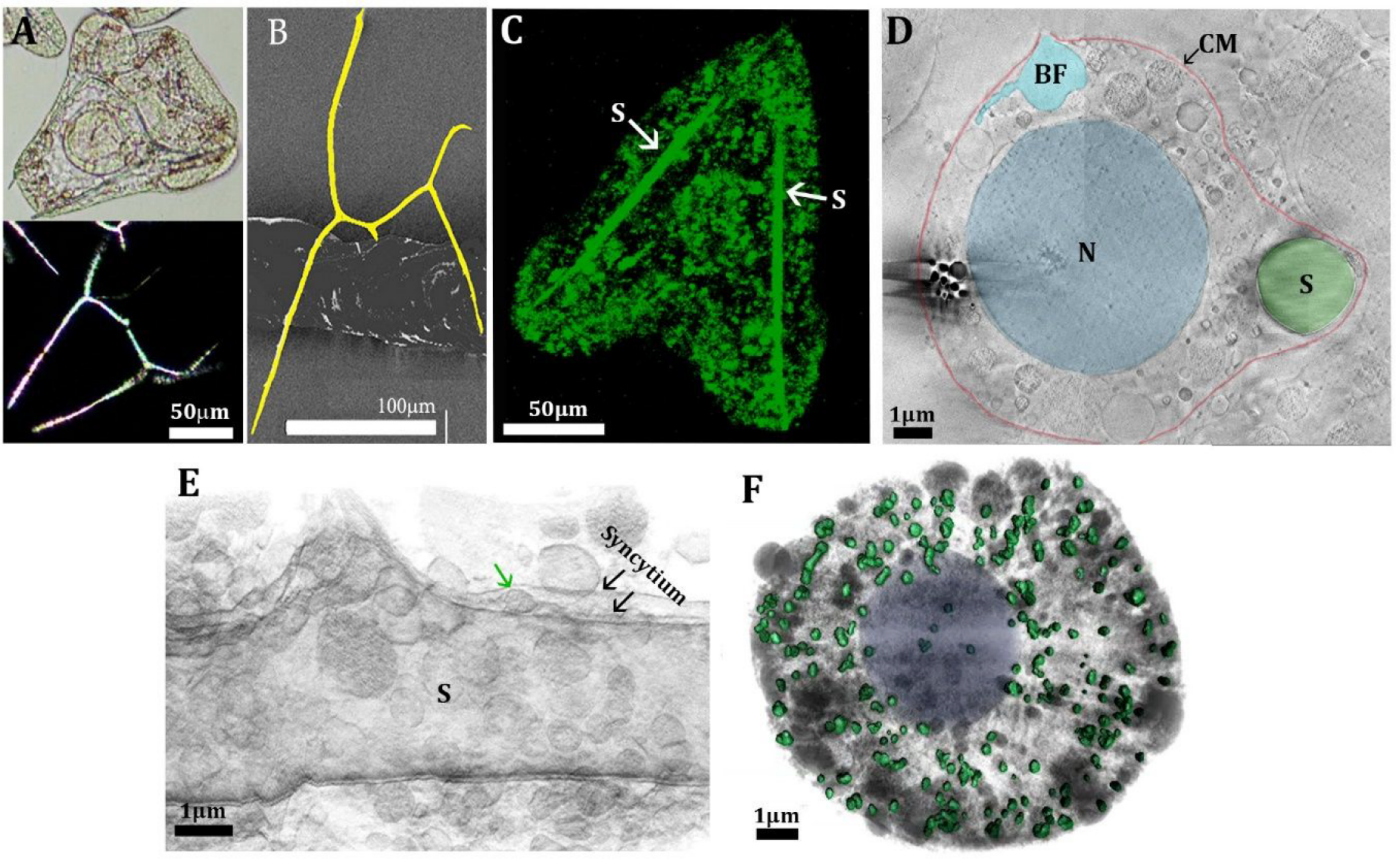
Mineral deposition process in sea urchin larvae. (A) Light micrographs of a live larva of the sea urchin *Paracentrotus lividus* taken 41 h after fertilization (hpf) (top image); image at the bottom: the same larva observed under polarized light, showing the crystalline nature of the spicules. (B) SEM micrograph of an isolated spicule from *Litechinus pictus* larva. The spicule is pseudocolored yellow to facilitate observation. (C) Confocal fluorescence image of a 46hpf larva developed continuously in calcein-labeled seawater. Note that the spicule (S) is fully labeled. Many intracellular vesicles are also fluorescent, indicating uptake by endocytosis of seawater. (D) Cryo-FIB-SEM micrograph of a high-pressure frozen 40hpf larva. The cyan vesicle is open toward the body cavity (blastocoel), which is filled with a seawater-like solution. BF, blastocoel fluid; CM, cell membrane; N, nucleus. (E) Segmentation of cryo-FIB-SEM serial milling and block face imaging stack acquired from 40hpf high-pressure frozen larva. The segmentation shows the syncytium enveloping the spicule. A vesicle (green arrow) is depositing onto the growing spicule. Reprinted and modified with permission from (41). Copyright 2016 Elsevier. (F) Segmentation of cryo-soft X-ray tomography of a PMC taken from 36hpf larva. The colored particles are Ca-rich particles. Cytoplasm and other cellular vesicles and organelles are gray.

Sea urchin larvae build a calcitic skeleton consisting of two ∼70 μm long and ∼3 μm thick spicules with convoluted morphology (Figure 2A,B). Interestingly, the fundamental characteristics of the spicule morphology remained essentially unchanged in sea urchin species over hundreds of millions of years.^28^ Spicule formation occurs inside a continuous membrane-bound volume (syncytium) that is formed by the fusion of cell membranes from specialized primary mesenchymal cells (PMCs), the cells that control spicule formation and mineralization.^29^ Each spicule diffracts X-rays or electrons as a slightly disordered single crystal of Mg-containing calcite, assuming the formula Ca_(1–*x*)_ Mg_*x*_CO_3_.^30^ The spicule mineral contains 3–5 mol % Mg^2+^ ions,^30c,31^ causing a minor reduction in the calcite crystallographic unit cell dimensions and an increase of 1.5% in solubility (log *K*_sp_ = −8.35) relative to pure calcite (log *K*_sp_ = −8.48).^32^ The reduction in the calcite cell dimensions indicates that at least part of the Mg ions substitute for Ca ions in the crystal lattice. The initially formed triradiate spicule, however, comprises 90% amorphous calcium carbonate (ACC) and 10% calcite and in its mature stage still contains >40% ACC.^33^ The ACC may accommodate more Mg relative to crystalline calcite, and thus, it may be expected that the Mg distribution is not homogeneous.^34^

#### Uptake and Transport

The source of calcium ions for spicule formation is seawater.^35^ Carbonate either comes exogenously from seawater or is produced endogenously through oxidative processing by the cells during respiration.^36^ Seawater enters the internal cavity of the sea urchin embryo, the blastocoel, which has a composition very similar to that of seawater.^37^ From the blastocoel, the ions enter the cells. When the seawater is spiked with the water-soluble and membrane-impermeable fluorescent dye calcein, all the cells, including both PMCs and the epithelial cells delimiting the larva, become fluorescent^19a,38^ (Figure 2C). The forming spicule emits fluorescence as well. The presence of calcein inside the cells indicates endocytosis of seawater, meaning that the calcein molecules are internalized together with the seawater in which they are dissolved.^19b^ Cryo-electron microscopy imaging showed that vesicles and vacuoles with diameters of 2 μm or even more are present within a PMC.^19a^ Some of the vacuoles have small openings to the blastocoel and could therefore take up seawater (Figure 2D). Some of these vacuoles are connected into networks of vesicles of variable sizes, ranging from few hundreds of nanometers to 1 μm. Thus, seawater and its contents can be distributed within this vacuolar network around the PMC. Calcium transport inside the cell and cell organelles may in part be mediated by calcium channels and/or calcium pumps. Calcium pumps are responsible mainly for the active transport of calcium out of the cell, but they are involved also in the uptake of calcium, especially in mitochondria.^39^ It does appear, however, that most of the calcium, if not all, originates from endocytosis.

#### Intracellular Ion Trafficking

Particles of amorphous calcium carbonate (ACC) form within intracellular vesicles in the PMCs.^40^ These ACC particles are eventually transferred to the syncytium and from there to the growing spicule^41^ (Figure 2E). Kahil et al. mapped the distribution of states and the concentrations of calcium in particles within PMCs that can be detected by cryo-soft X-ray spectroscopy.^40b^ There are around 200 Ca-containing particles in one cell (Figure 2F). The size of the Ca-containing particles ranges from 100 to 500 nm, and the Ca concentration inside the particles ranges from 1 to 15 M. The chemical environment of the Ca ions ranges in a continuum from calcium in a concentrated water solution to calcium in anhydrous ACC. The particles may be occluded individually inside a vesicle, or there may be multiple particles in one vesicle. Kahil et al. surmise that calcium from seawater concentrates gradually inside the vesicle, until particles of ACC precipitate.

Several other processes must, however, be performed before the ACC particles condense and move to the spicule. In seawater, the ratio of Ca to Mg is 1:5, and in the spicule mineral the Mg content is 5 mol %. In seawater, Na and Cl dominate and they are practically absent from the spicule mineral. This means that a major part of the Mg ions and all Na and Cl ions must be removed from the Ca-containing vesicles. How this occurs is not known, but Mg, Na, and Cl can conceivably be released into the cytosol environment as free or complexed ions and/or trafficked outside the cell by transporters.^42^ Magnesium is involved in the regulation of several cellular functions, and major fluxes of Mg ions in either direction occur across the plasma membrane.^43^ But how cells regulate Mg^2+^ homeostasis and transport is far from clear.^44^ Na and Cl ions are mainly involved in osmotic pressure regulation. Sodium ions exchange with potassium ions, whose concentration is in turn regulated through potassium ion channels. Chloride ions also exit cellular membranes through selective chloride channels.^45^

The calcium counterion in the mineral phase, carbonate, is present in seawater at 0.3 mM concentration, which combined with the calcium concentration already exceeds calcite or ACC solubility.^46^ This means that calcium carbonate mineral in theory can precipitate from the endocytosed water using only the Ca and carbonate ions that are there, provided that nucleation inhibitors such as Mg have already been removed. Even more carbonate ions become available, if the ten-times more abundant bicarbonate ions transform into carbonate, with subsequent release of protons. PMCs use Na^+^/H^+^ exchange mechanisms to control cellular pH homeostasis during maintenance of the skeleton.^36b^ Recently, a new transporter was identified, which mediates the exit of protons from the cell.^47^ It is not clear whether the same transporter also mediates exit of protons from the mineralizing vesicles. The enzyme carbonic anhydrase catalyzes the transformation of carbonic acid into protons and bicarbonate ions. Three carbonic anhydrase enzymes specific to PMCs, have been identified.^48^ It is likely that carbonic anhydrase contributes to the regulation of carbonate homeostasis, shifting the equilibrium in the direction of carbonate production, when protons are removed. Additional sources of carbonate may be carbonate transporters from the blastocoel, or synthesis from the oxygen processed during respiration in the mitochondria.

#### Mineralized Tissue Formation

Even though each spicule is a single calcite crystal,^30a−30c^ more than one calcite nucleation site exists inside the syncytium in the first stages of spicule deposition.^49^ The different nucleation sites compete with each other, until only one nucleation site prevails. A calcite crystal forms with distinct morphology and grows into a planar triradiate spicule. The arms of the triradiate spicule extend along the a* crystallographic axes of calcite, and the calcite *c* axis is perpendicular to the triradiate spicule. In a later stage, the crystal elongates along the *c* axis, giving rise to the body rod, and subsequently to the other rods, which complete the convoluted spicule morphology (Figure 2B).^50^ How the relation between the crystallographic directions of calcite and the directions of growth of the spicule inside the syncytium is controlled and regulated by the cells is one of the unsolved mysteries of sea urchin larval spicule growth.^51^

Amorphous calcium carbonate particles, formed inside the PMC cells, are extruded into the syncytium (Figure 2E). Here, they attach both to the rod extremities to elongate the growing spicule and along the length of the rods to thicken them. Once the newly extruded particles join with the already crystallizing spicule, they crystallize by secondary nucleation in a manner akin to percolation.^52^ Before and during this stage, considerable amounts of water must still be eliminated from the particles, because crystalline calcite does not contain lattice water. Not all the particles, however, crystallize,^33,53^ and the intimate coexistence of the crystalline and amorphous material probably contributes to reducing spicule brittleness and increasing spicule flexibility.^54^

An additional parameter contributing to structuring the spicule is the glycoproteins composing the organic matrix of the spicule. There is little organic material inside the spicule (0.05% of mineral), and this includes about 50 proteins that have been identified, and form the protein matrix of the spicule.^55^ These proteins are most probably responsible for many characteristics of the mature spicule, including the nucleation site, the growth into the complex morphology, and contribute to the mechanical properties. Little however is known about the protein functions.^56^

### Vertebrate Bone

**Figure 3:**
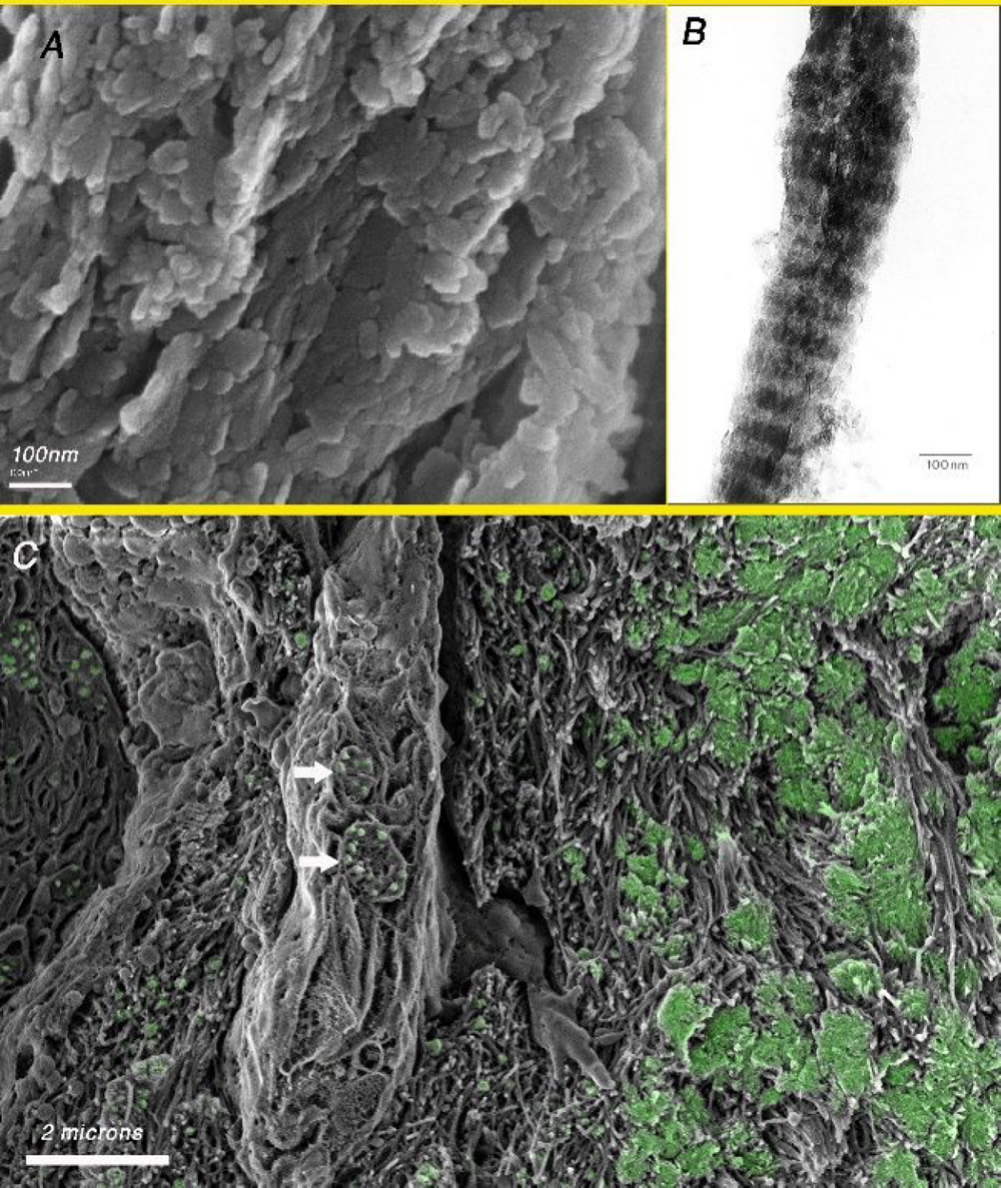
(A) SEM image of fractured baboon tibia after removal of the organic matrix using sodium hypochlorite. Note the plate-shaped crystals organized in layers. (B) TEM image of an isolated mineralized collagen fibril extracted mechanically from turkey tendon. The banding is due to the presence of more plate-shaped crystals in the gap region of the collagen fibril as compared to the overlap region. (C) Cryo-SEM image of the fracture surface of an embryonic chicken bone showing in green the distribution of mineral (based on the BSE image of the same area). Note the presence of vesicles containing mineral particles inside the cell (arrows).

The mineralized collagen fibril is the building block of bone (Figure 3A).^57^ The plate-shaped crystals are aligned in layers across the fibril, and as the fibrils themselves are aligned, the overall structure is layered (Figure 3B). The collagen fibrils can be arranged in different ways to form different bone types.^58^ Bone is thus a composite of mainly organized arrays of Type I collagen fibrils impregnated and surrounded by plate-shaped crystals of carbonate hydroxyapatite, whose average composition for one specific bone is (Ca_8.54_Mg_0.25_Na_0.38_)[(PO_4_)_4.99_(CO_3_)_1.01_](OH)_0.99_.^59^ The crystals are about 250 × 500 × 2–4 nm in dimensions^1^ (Figure 3B). In bone mineral, carbonate constitutes about 4–6% by weight.^60^ Carbonate plays a crucial role in the mineral formation processes, such as the determination of crystal shapes and the stability of the crystals.^61^

There are clearly parallel and/or alternative calcium, phosphate and carbonate pathways involved in the formation of bone mineral. This complexity/redundancy in part may reflect the fact that bone formation is a relatively well investigated system. The complexity may also be a strategy that is tailored to meet different requirements, such as rapid surface thickening of bone, especially during early development, versus maintenance of mature bone by remodeling.^62^ Furthermore, there are pathways that are specific to one process, such as bone elongation in the growth plate, or pathways that are responsible for producing hyper-mineralized bones.^63^ It is therefore challenging to identify common underlying ion pathways in bone formation.

#### Uptake and Transport

The ultimate source of ions for bone mineralization is the environment, i.e., the food and ions in water for aquatic vertebrates and food and drinking water for terrestrial vertebrates. Uptake of ions occurs in the intestinal tract. These ions are transferred in solution through the walls of the tract into the vasculature.^64^ Many fish deposit amorphous calcium carbonate in their intestinal tracts, often in large quantities.^65^ It has been suggested that the amorphous phase is produced in order to store precipitated CaCO_3_ temporarily in the intestine.^65b,66^ Many herbivores form spherulites of calcium carbonate in their intestinal tracts.^67^ So these fish and herbivores are able to extract more calcium and carbonate in their intestines than they need for their metabolic purposes and for skeleton formation.

Calcium, carbonate, and phosphate are transported in the vasculature. Most mammalian biofluids (blood serum, saliva, etc.) are supersaturated with respect to carbonate hydroxyapatite, and when a precipitate forms, the phase that forms is amorphous calcium phosphate (ACP).^6b^ An interesting observation reported by Neuman and Neuman is that “an isolated sample of blood or serum can ‘hold’ considerably greater quantities of calcium and phosphate than occur normally.”^6a^ This is presumably due to the fact that many of these ions are bound to charged molecules and macromolecules, and especially phosphopeptides that form a stable complex with ACP.^6b^ Holt et al. suggest that this allows most biofluids to remain stable near physiological pH, even though they are supersaturated with respect to carbonate hydroxyapatite. This in turn enables hard and soft tissues to coexist. Blood is replete with potent crystal nucleation inhibitors that prevent precipitation, such as Matrix Gla Protein (MGP),^68^ fetuin-A,^69^ and polyphosphate.^70^ Removal of these inhibitors results in catastrophic ectopic mineral formation. Vertebrates have thus evolved a widespread system of inhibitors that have to be removed at the site of mineral deposition in order to enable skeletal formation (see below).

Vertebrates use the mineral in many of their bones as a reservoir to maintain ion homeostasis (reviewed in ref (71)). The short-term maintenance of calcium and phosphate concentrations in the serum is achieved via the cells embedded in bone and the thin channels that connect them (the osteocyte-canalicular system).^72^ The long-term maintenance of calcium and phosphate levels is by removal of small volumes of bone by osteoclasts and deposition of new bone by osteoblasts in the remodeling process.

Another strategy used for preventing ectopic mineralization is to transport solid mineral in the blood within membrane-bound vesicles, as was observed in chick embryos.^73^ Such membrane-bound vesicles were only observed in sparse amounts in the growth plates of 9 week old mice.^74^ The structure of these mineral containing vesicles in blood serum and in osteoblasts and osteoclasts is very similar, namely, small mineral particles in a relatively large vesicle^73,75^ (Figure 3C). This raises the possibility that these mineral-containing vesicles are derived from bone that has been removed by osteoclasts and the vesicles with their mineral particles are transported to the osteoblasts in the blood serum.

#### Intra- and Intercellular Ion and Mineral Trafficking for Bone Formation and Resorption

Osteoblasts are responsible for bone extracellular matrix (ECM) formation and mineralization. The osteoblasts presumably receive ions and mineral from the vasculature.^76^ These ions are temporarily stored within the cell in various organelles, including the membrane-rich endoplasmic reticulum and the mitochondria. Solid mineral deposits are frequently observed in mitochondria.^77^ Vesicles containing small granules of mineral have also been identified in osteoblasts^75b,78^ (Figure 3C). Some of these vesicles are closely associated with the mitochondria.^79^ The mineral phase within these vesicles is disordered calcium phosphate (ACP).^75b^

Some of the intracellular ions in osteoblasts are used for maintaining homeostasis within the cells, whereas most of the soluble ions are transported into the mineralizing ECM. In some cases, a solid ACP phase is transferred into the mineralizing ECM.^80^ A third route for transferring ions into the mineralizing ECM is via the budding of vesicles that have the capability of taking up soluble ions from the extracellular environment and depositing them as an amorphous phase.^81^ These are the so-called matrix vesicles.^82^ As not all mineralizing tissues seem to have solid mineral particles or produce matrix vesicles, we infer that the major intracellular pathways for mineralization involve dissolved and/or bound ions of phosphate, calcium and carbonate.

In zebrafish tail bone formation, Akiva et al. observed that the vast majority of cells present around the forming tail bone do not have intracellular mineral particles.^83^ Furthermore, the cells maintain a thin space between them that is continuous between the artery and the forming bone.^84^ Akiva et al. therefore raised the possibility that ions in solution or ions bound to other molecules may be transported directly from the vasculature to the site of bone mineral formation without entering cells.

During long bone elongation, hypertrophic cartilage forming cells (chondrocytes) produce an extracellular collagen and proteoglycan rich matrix. This matrix mineralizes at some distance from the cell surface.^85^ The hypertrophic chondrocytes do not form any intracellular solid mineral.^85b^ It is therefore inferred that the source of the ions for cartilage mineralization is directly from the blood vessels.^74,85b^

#### Mineralized Tissue Formation

The preformed organic matrix of bone is dominated by fibrils composed of Type I collagen molecules. The first mineral that is deposited in this matrix forms isolated islets of amorphous ACP^86^ (Figure 3C), which in osteoblast cell cultures is carbonate rich.^61b^ In the zebrafish tail fin, these islets of ACP are located between fibrils.^80^ The mineral subsequently penetrates into the fibrils where crystals of carbonate hydroxyapatite are nucleated and grow first in the gap zone as needles.^87^ The crystals then become plate-shaped and subsequently penetrate into the overlap zones.^88^ In many bones, crystals are also observed to be arranged on and/or close to the surface of the fibrils.^89^

There are many other macromolecules (proteins, proteoglycans and glycoproteins) in this matrix, but not inside the collagen fibrils. There is an intricate interplay between the Type I collagen structure and the local ionic strength that is controlled mostly by the proteoglycans.^90^ For example, in the preformed matrix of the continuously forming incisor of a rodent, the proteoglycan assemblage and the collagen fibril morphology changes from close to the cell surface to the mineralization front.^91^ In parallel the collagen fibril structure changes, as evidenced by an increase in the fibril diameter, and a change in the axial repeat structure (D-banding) from 67 to 71 nm.^92^ The deposition of the initial mineral in the organic matrix takes place in these extended and swollen fibrils. It is conceivable that these structural changes facilitate the ACP penetration into the fibril gap zones. It has also been observed that the C-terminal ends of the fibrils promote the infiltration of the mineral, and the charged residues in the gap and overlap regions induce crystallization of the amorphous phase.^93^ Another implication of this process is that the formation of crystalline carbonate hydroxyapatite has to initially be prevented outside the collagen fibril, and this requires the presence of crystal inhibiting molecules, such as fetuin or pyrophosphate.^94^

## Discussion

**Figure 4:**
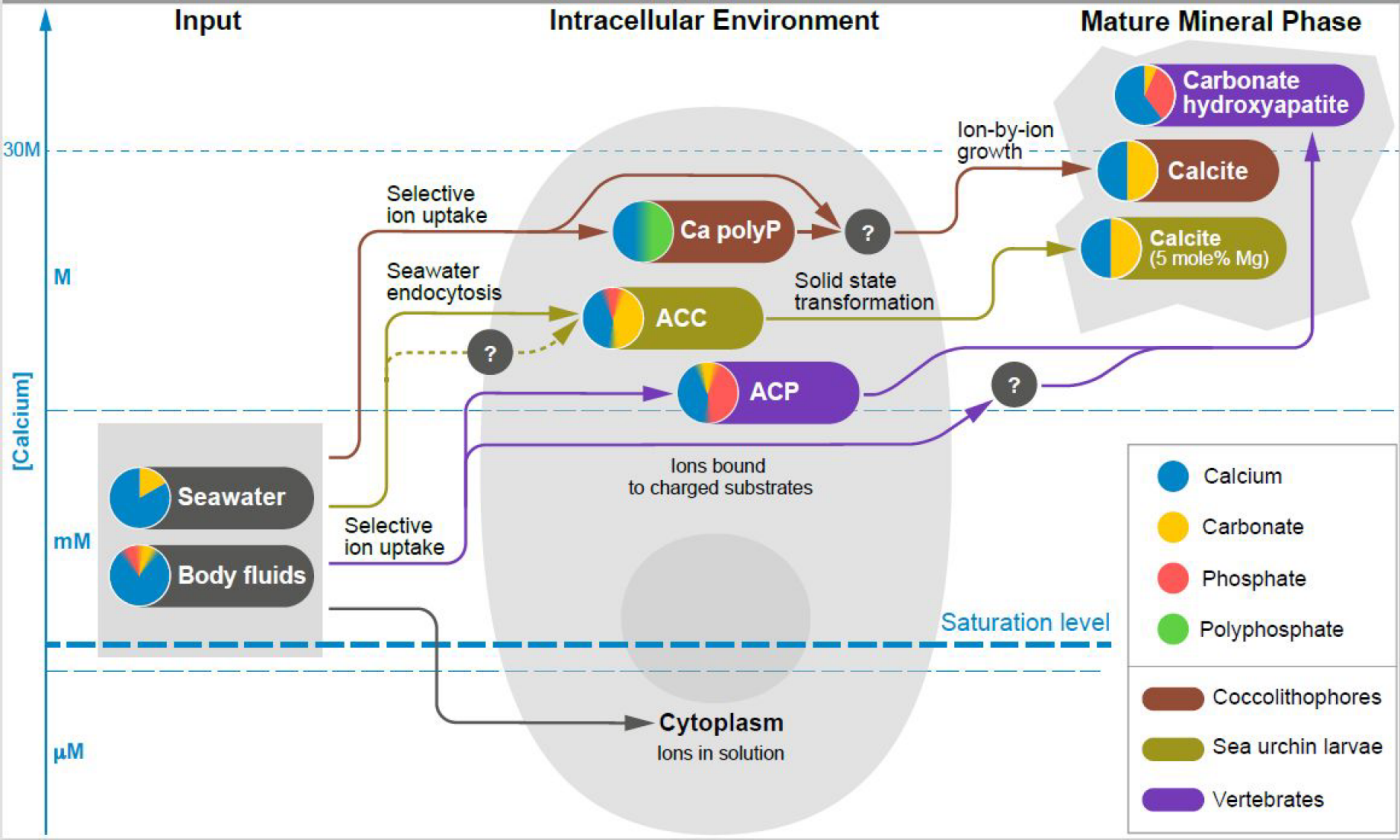
Schematic illustration showing the three common processes that make up ion pathways in the biomineralization of coccolithophores, sea urchin larvae, and vertebrate bone. These pathways involve ion uptake, cellular manipulation and deposition of the mature mineral phase. The *Y*-axis shows the approximate calcium concentration ranges in which these three processes operate. The solid lines indicate known processes and dotted lines indicate putative ones. Question marks highlight yet uncharacterized stages in the pathway. Note the enormous calcium concentration range for mineralizing cells. Irrespective of whether the mature mineral phase is a carbonate or phosphate mineral, the ion pathways all involve calcium, carbonate and phosphate as is schematically illustrated by the blend of colors for the different ions.

At first sight, the test cases described above may give the impression that evolution gave rise to diverse systems, where each organism uses a very specific set of tools and pathways to form minerals. In some aspects, such as the protein complexes, the functional macromolecules and even the mineral composition, this is correct. The three test cases do however share common traits, such as forming intracellular mineral deposits isolated from their environments by membranes, manipulating solutions that are saturated with respect to calcium carbonate and/or calcium phosphate, and the involvement of different ions, such as carbonate in phosphate minerals and polyphosphate in carbonate minerals. Significantly, the chemical nature of the mineralization process imposes the same set of physical limitations on each of the biological systems. By integrating the chemical processes and the properties of the chemical phases, we can derive some insights into the pathways, which evolved to circumvent the chemical constraints that would prevent the material from fulfilling its biological function. These points are schematically illustrated in Figure 4.

A starting point is to consider the nature of the fluids involved in biomineralization and their common solution chemistry properties. A common trend that can be deduced from the test cases is that most, if not all, the aqueous solutions are supersaturated with respect to at least one of the relevant mineral phases. In vertebrates, the intestinal tract, the blood, the extracellular space in forming bone, and the environment within the collagen fibrils are all supersaturated with respect to at least one phase of calcium phosphate.^4c,95^ Seawater is supersaturated with respect to calcite,^5^ and so is presumably the extracellular space of the sea urchin larval spicule and the coccolith vesicle, where calcite precipitates. It is therefore interesting to consider that one of the main chemical properties that needs to be controlled along the crystallization pathway is the inhibition of nucleation of unwanted mineral phases prior to the precipitation at the final site. In vertebrates, many macromolecules that inhibit nucleation in vivo have been identified.^68,69,76^ It would not be surprising if analogous nucleation inhibiting systems involving macromolecules are active in sea urchins and coccolithophores.^96^ There are many examples of proteins that inhibit nucleation in vitro.^93,97^

It seems that the common chemical mechanism for inhibiting nucleation from seawater and body fluid solutions is the presence of ionic inhibitors, which operate on the kinetics of the crystallization rather than on the thermodynamics, making the formation of crystal nuclei improbable. A good example is the high concentration of Mg ions in seawater that prevents nucleation of calcium carbonate.^5^ Mg may have a similar role in the blood serum. It is interesting to note that many pathological minerals found in humans contain Mg,^98^ indicating that Mg is a priori present and when uncontrolled precipitation occurs Mg is incorporated into the ectopic solid phase. Solutions rich in many nucleation-inhibiting chemical species make it possible to achieve high degrees of supersaturation and molecular crowding. These are also the conditions that favor the initial formation of amorphous phases rather than the more stable crystalline phases (Ostwald’s Rule of Stages^99^). Accordingly, the common biological strategy of using intermediate amorphous phases as precursors for mineral formation, may well have evolved from the common need of organisms to prevent ectopic nucleation, and by so doing facilitate the formation of metastable solutions.

One fundamental enigmatic issue for all three test cases is understanding the factors that control the ordered nucleation of the final mineral phases. Nucleation does not occur just because of removal of inhibitory factors, but is rather controlled in space, in time and along the sequence of events. In several cases in biomineralization, acidic macromolecules adsorbed on a nucleating surface were shown to induce oriented nucleation of crystals by virtue of their complementarity to ionic motifs on specific crystal planes.^100^ Interestingly, the same complementarity may lead to inhibition of crystal nucleation or growth when the macromolecules are not immobilized on the nucleating surface,^97b,101^ providing further protection against ectopic nucleation.

In all three test cases examined here, some kind of dense amorphous phases were observed, even though the Ca-rich bodies within coccolithophores are intracellular storage sites that do not participate directly in the crystallization process. For sea urchin larval spicules and bone, the crystallization mechanisms by which the mature crystalline phase is formed involve a direct solid-state transformation of an amorphous precursor, whereas for coccoliths the process includes ion-by-ion crystal growth. In principle, any intermediate between ion-by-ion crystal growth and crystallization from an amorphous precursor is conceivable, and there are probably processes in biomineralization covering the whole range between the two options.^102^ In the sea urchin, the ACC granules crystallize by secondary nucleation from one original oriented crystal located in the center of the triradiate spicule.^97d,103^ During bone formation, ACP particles penetrate the collagen fibril and give rise to the much smaller oriented carbonate hydroxyapatite crystals nucleated within the collagen fibrils. The charged residues in the gap zone of collagen may be the nucleating agents, in the absence of an additional nucleating protein.^104^ The coccolith crystals nucleate in close proximity to an organic substrate and grow inside the coccolith vesicle in a process that resembles ion-by-ion growth.^10^

A general principle in biologically controlled crystallization is that biology achieves control over phase transitions by reducing extreme jumps in energy levels. Nucleation is defined as a critical phenomenon, which occurs catastrophically at high supersaturation, once the activation energy barrier is overcome. By controlling the chemical environment of the nucleation sites through compartmentalization and control over pH and/or by introducing inhibitors and catalysts, the energy barriers are reduced, creating smoother energy landscapes. This is a general concept in biology, which is clearly relevant in biomineralization.

The presence of various intermediate mineral phases in organisms that employ different crystallization pathways suggests that crystallization via an amorphous precursor is not necessarily a linear process of sequential phase transitions that ultimately form the mature mineral. It appears to be more complex and flexible, as dense intracellular and extracellular mineral phases can serve diverse functions, from storage or removal to crystallization. This attribute is shared by all three test cases. Different dense mineral phases are present in the coccolithophore cell and involve polyphosphate complexes, whereas the final product is calcium carbonate. This situation is similar in sea urchin larval PMC cells, where some new evidence indicates that relatively large amounts of phosphate ions may contribute to the ACC phase stability. In the vertebrates, the variety of dense mineral phases produced, with carbonates in the calcium phosphate minerals and possibly phosphates in the carbonate minerals, may point to different functions for these minerals. One example is the formation of a disordered precursor phase with a Ca/P ratio of around 1.^105^ Other examples include the formation of amorphous calcium carbonate in the intestinal tract of fish and calcite in the vestibulary system.^65^

An important compositional change that is shared by all three cases is the need to exclude from the initial amorphous phases chemical species that are abundant in the mother solutions and are absent or almost absent in the mature mineral phase. The most abundant component of mother solutions is water, and the transient amorphous phases are still highly hydrated. Mg ions, and especially sodium and chloride ions, which are abundant in seawater, are a minor constituent in coccolith and sea urchin calcite. How this is achieved is not clear at all. Also some macromolecules that interact with the mineral ions in intermediate stages need to be excluded, for example, polyphosphates that complex calcium in coccolithophores and fetuin, pyrophosphate, and polyphosphate in vertebrates. Here, the marked differences between the test cases may point to important disparities. The calcite of coccoliths is very pure, both in terms of inorganic and macromolecular impurities. This may result either from a very selective transport process into the coccolith vesicle, or from the chemical process of mainly ion-by-ion growth that more easily enables exclusion of impurities. On the other hand, the sea urchin spicule calcite contains about 0.1% by weight macromolecules and can incorporate approximately 5 mol % MgCO_3_. This may stem from the direct incorporation of solution that originates from seawater and the solid-state transformation of the amorphous precursor phase. Pokroy et al.^106^ have demonstrated the presence of high and low Mg calcite phases in echinoderm skeletal elements that are thought to have been formed by spinodal decomposition.

From the biological side of the crystallization process, cells need to maintain homeostasis and this imposes chemical constraints. For example, the concentration of free calcium in the cytoplasm cannot exceed the micromolar level. Some organisms solve this problem by enabling the formation of calcified minerals via extracellular transport routes, as may occur in some bone formation processes. Another option is to keep the intracellular Ca-rich phases compartmentalized along the crystallization pathway, as occurs in all three test cases. It is still unclear how coccolithophores maintain the needed flux of calcium, as the coccolith vesicle is intracellular and not in direct contact with any seawater-derived solution. Since intracellular ion-rich phases can be found in all three cases, as well as in many other organisms, some of which do not form any kind of minerals, it seems that the ability to concentrate ions inside a cell is rooted deep in evolution and represents a common feature of many organisms.

## Concluding Comment

Organisms build mineralized materials with unique mechanical-structural properties and architectures, often with a hierarchical complexity well above that of our most advanced synthetic materials. In most cases, we do not know how this is achieved, to the point that it may appear that biology bends the rules of chemistry and thermodynamics to achieve what it does. This is obviously not true. Only recently are we starting to understand some of the rules of the game, as well as some of the fundamental problems that biology is faced with when building mineralized materials starting from relatively dilute ion solutions.

We understand that even though biology cannot bend the rules of chemistry, it can put chemistry at the service of biomineralization by manipulating the environment, the conditions, the components, and the kinetics of the system. The rules and problems that we identify here may apply to many more mineralization processes than the three test cases examined here.

Were we to try to understand the ion pathways that organisms evolved for uptake, transport, and deposition of calcium, carbonate, and phosphate ions, using only the tools of synthetic chemistry, we would inevitably fail. The only way to really understand these complex pathways is to always keep in mind that indeed biology is chemistry with a history.^3^
